# Clearing muddied waters: Capture of environmental DNA from turbid waters

**DOI:** 10.1371/journal.pone.0179282

**Published:** 2017-07-07

**Authors:** Kelly E. Williams, Kathryn P. Huyvaert, Antoinette J. Piaggio

**Affiliations:** 1 USDA, Wildlife Services, National Wildlife Research Center, Wildlife Genetics Lab, Fort Collins, Colorado, United States of America; 2 Department of Fish, Wildlife, and Conservation Biology, Colorado State University, Fort Collins, Colorado, United States of America; 3 School of Environmental and Forest Sciences, University of Washington, Seattle, Washington, United States of America; University of Hyogo, JAPAN

## Abstract

Understanding the differences in efficiencies of various methods to concentrate, extract, and amplify environmental DNA (eDNA) is vital for best performance of eDNA detection. Aquatic systems vary in characteristics such as turbidity, eDNA concentration, and inhibitor load, thus affecting eDNA capture efficiency. Application of eDNA techniques to the detection of terrestrial invasive or endangered species may require sampling at intermittent water sources that are used for drinking and cooling; these water bodies may often be stagnant and turbid. We present our best practices technique for the detection of wild pig eDNA in water samples, a protocol that will have wide applicability to the detection of elusive vertebrate species. We determined the best practice for eDNA capture in a turbid water system was to concentrate DNA from a 15 mL water sample via centrifugation, purify DNA with the DNeasy *mericon* Food kit, and remove inhibitors with Zymo Inhibitor Removal Technology columns. Further, we compared the sensitivity of conventional PCR to quantitative PCR and found that quantitative PCR was more sensitive in detecting lower concentrations of eDNA. We show significant differences in efficiencies among methods in each step of eDNA capture, emphasizing the importance of optimizing best practices for the system of interest.

## Introduction

The need for more effective ways to assess biodiversity and to detect and monitor invasive or endangered species has fueled the advancement of methods for identifying DNA shed from an organism into the environment: environmental DNA or eDNA [1–4]. Successful implementation of eDNA-based detection requires successful capture of eDNA, even when it may be in low concentrations in the environment [2, 5–8]. Water samples may contain varying amounts of target DNA due to several factors including the size of the shedding organism, the volume and intensity of secretion or shedding, and the rate of degradation of eDNA in the water [9–13]. Further, abiotic features of aquatic systems including turbidity, temperature, pH, size of water body, and flow rates can affect persistence of the target eDNA [14]. Beyond the challenges posed by capture of eDNA, laboratory purification and successful amplification of the target region(s) can be difficult. Each step requires optimization for sensitivity and specificity, along with sustained efforts to monitor for and minimize contamination because eDNA samples are inherently of low quality and low quantity.

Multiple methods are available for each step in the capture (concentration), purification (extraction), and amplification steps for eDNA. Most eDNA studies have been performed in clear, marine [8, 15, 16] or freshwater systems [2, 5, 6, 13, 17, 18] but the detection of terrestrial invasive and/or endangered species may rely on sampling at intermittent water sources that are used for drinking and cooling which may be small, stagnant, or turbid. Turbid water poses a unique set of challenges to detect eDNA because the eDNA can be absorbed in soils and sediments by the formation of cationic bridges between the phosphate groups of the nucleic acids and clay surfaces, protecting the DNA from degradation [19–21]. Stagnant water may also contain inhibitors, which are humic substances that, when co-extracted with DNA, can interfere with PCR amplification, making detection more difficult [22–24]. Our goal in this study was to determine the best practices for capture, purification, and amplification of eDNA shed from an invasive, terrestrial mammal (feral pigs, *Sus scrofa*) into turbid water.

### Feral pigs

Feral pigs are considered one of the most widespread, invasive vertebrate species worldwide [25, 26]. Due to the negative impacts of feral pigs on agricultural and natural ecosystems and their continued expansion, management efforts are being focused on reducing feral pig populations [27]. Major challenges to eradication involve detecting the last few individuals during an eradication effort, new invaders, or small populations transported by humans [25, 28–30]. An accurate, highly sensitive method of detection of feral pigs is needed to help managers discover individuals when numbers are low and before the population increases to an unmanageable point.

Feral pigs drink or wallow in water [31, 32] during which they shed cells containing DNA. In some areas across this species’ range in the United States, such water sources are small, intermittent, stagnant, and turbid. An eDNA detection technique could provide an ideal approach for detection and monitoring of this invasive species by sampling water bodies.

### Approaches to detecting environmental DNA (eDNA)

**Fig 1:**
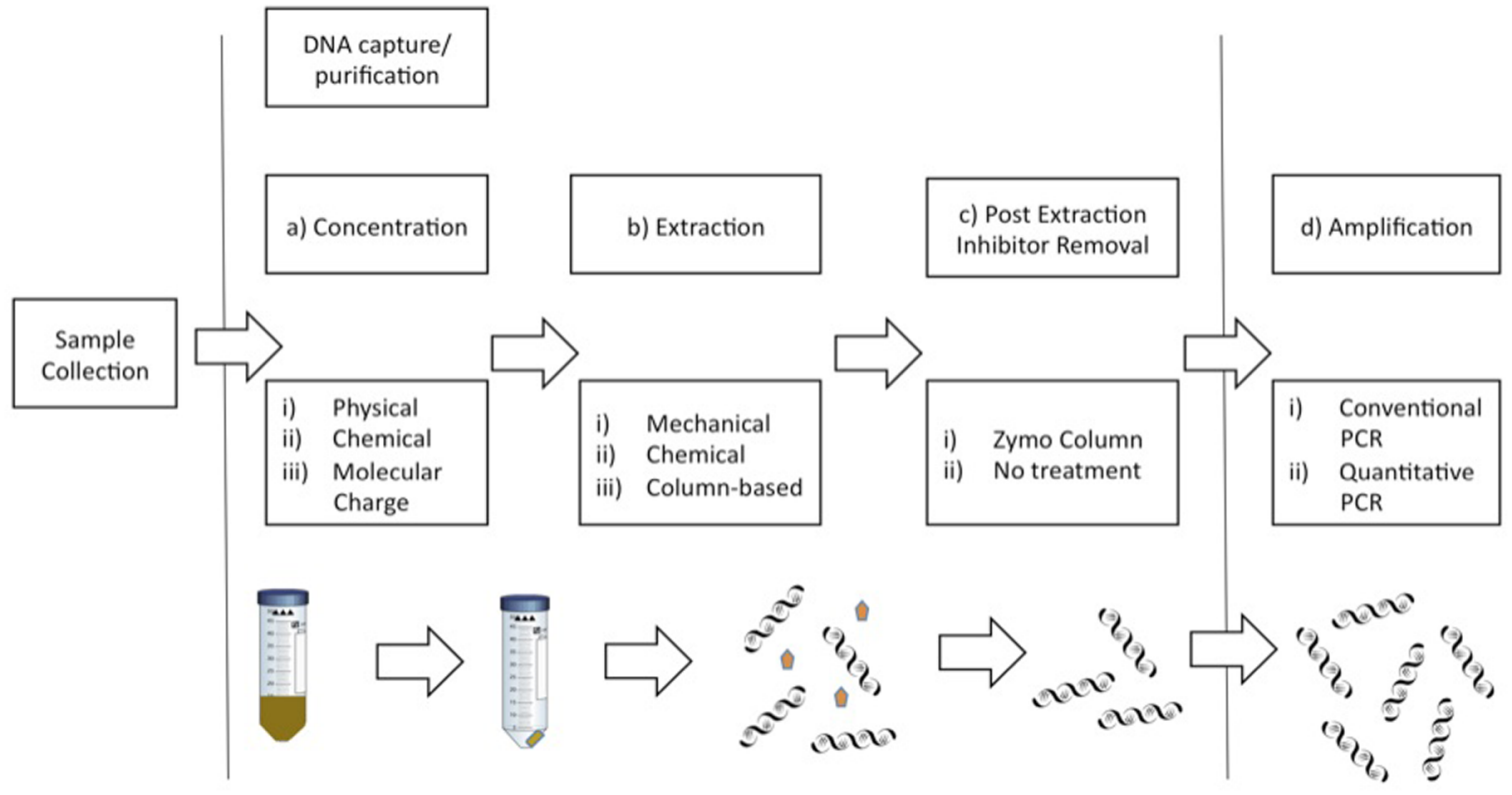
Process of eDNA capture involves concentration, extraction, inhibitor removal, and PCR amplification of a water sample.

Multiple approaches have been applied to the capture of eDNA from aquatic systems; we examined the efficiency at detecting eDNA using different combinations at each step of the process, detailed below and summarized in Fig 1. Concentration of DNA using precipitation with sodium acetate and ethanol [2, 33], centrifugation [15, 34], and filtration [5, 6, 35] have all been shown to successfully capture eDNA from bodies of water. Precipitation and centrifugation constrain the water sample volume but filtration methods often clog, hindering the capture of eDNA, particularly in turbid waters [18]. Resin beads have been used to capture virus particles in wastewater by offering an anionic exchange area and attracting particles with a negative surface charge [36]. Similarly, resin beads may allow for capture of small fragments of DNA from large quantities of water, which could be ideal for eDNA collection in the field. The comparison of each of these methods for eDNA capture in turbid waters has not been performed and was one objective of this study.

Each step has multiple options that are optimal for the system that the water is collected from. The concentration step (a) captures the eDNA into a form that extraction methods can work with (i.e. transformation of water to pellet in a centrifuge tube). The extraction method (b) then purifies the DNA captured in the pellet with varying efficiencies (curved lines represent DNA, pentagons are inhibitors). An inhibitor removal post-extraction step (c) may effectively remove inhibitors while also potentially causing a loss of DNA. Finally, either conventional PCR or quantitative PCR can be chosen for the amplification technique, which may differ in sensitivity to low quantities of DNA.

Extraction methods for purification also vary among eDNA studies. These methods exploit different techniques for purifying DNA including chloroform-based protocols [37–39], physical disruption [6, 40], and column-based techniques [2, 9, 18]. Different combinations of concentration and extraction methods also produce varied results [4, 41]. The choice of an optimal concentration and extraction combination is likely dependent on the aquatic system from which the eDNA sample is collected. Turbid water may require a different method for purifying eDNA compared to freshwater or marine systems; we tested multiple extraction techniques in combination with various concentration methods to identify the optimal protocol for samples collected from turbid waters.

An inhibitor removal step post-extraction can remove humic substances that may interfere with downstream PCR reactions [23, 24]; such a step may also help optimize eDNA detection from turbid waters. Coextraction of inhibitors is not a new challenge for the eDNA field. Multiple studies show inhibitors are present in water and soil eDNA extractions and pose a challenge to detection [42–45]. The trade-off of an inhibitor removal step post-extraction is the risk of losing some DNA in a sample that is already of low quantity potentially leading to a false negative result [46]. Diluting the sample can also dilute inhibitors but inhibitor removal columns are thought to be superior to dilution [24, 47]. To improve efficiency in capturing and detecting eDNA from turbid water in this study, we tested a kit-based inhibitor removal system (Zymo Research, Irvine, California, USA).

Lastly, amplification success of purified eDNA samples may vary depending on the PCR approach: quantitative PCR (qPCR) tends to be more sensitive for detecting eDNA than analysis by conventional PCR (cPCR) on a genetic analyzer or gel [48, 49]. Here, we compared the sensitivity of our feral pig eDNA assay across both platforms (fragment analysis with cPCR verses qPCR) to optimize the sensitivity of the assay for detecting low concentrations of eDNA captured from turbid water.

## Methods

Laboratory work was performed at the USDA-APHIS National Wildlife Research Center in Fort Collins, Colorado, USA. Extractions were performed in a lab where only low quantity/low quality DNA was processed. All PCR and post-PCR procedures were completed in separate rooms. To minimize contamination of the samples, equipment, benchtops, pipettors, and fume hoods were cleaned with a 10% bleach solution before and after any procedure.

### eDNA extraction

**Fig 2:**
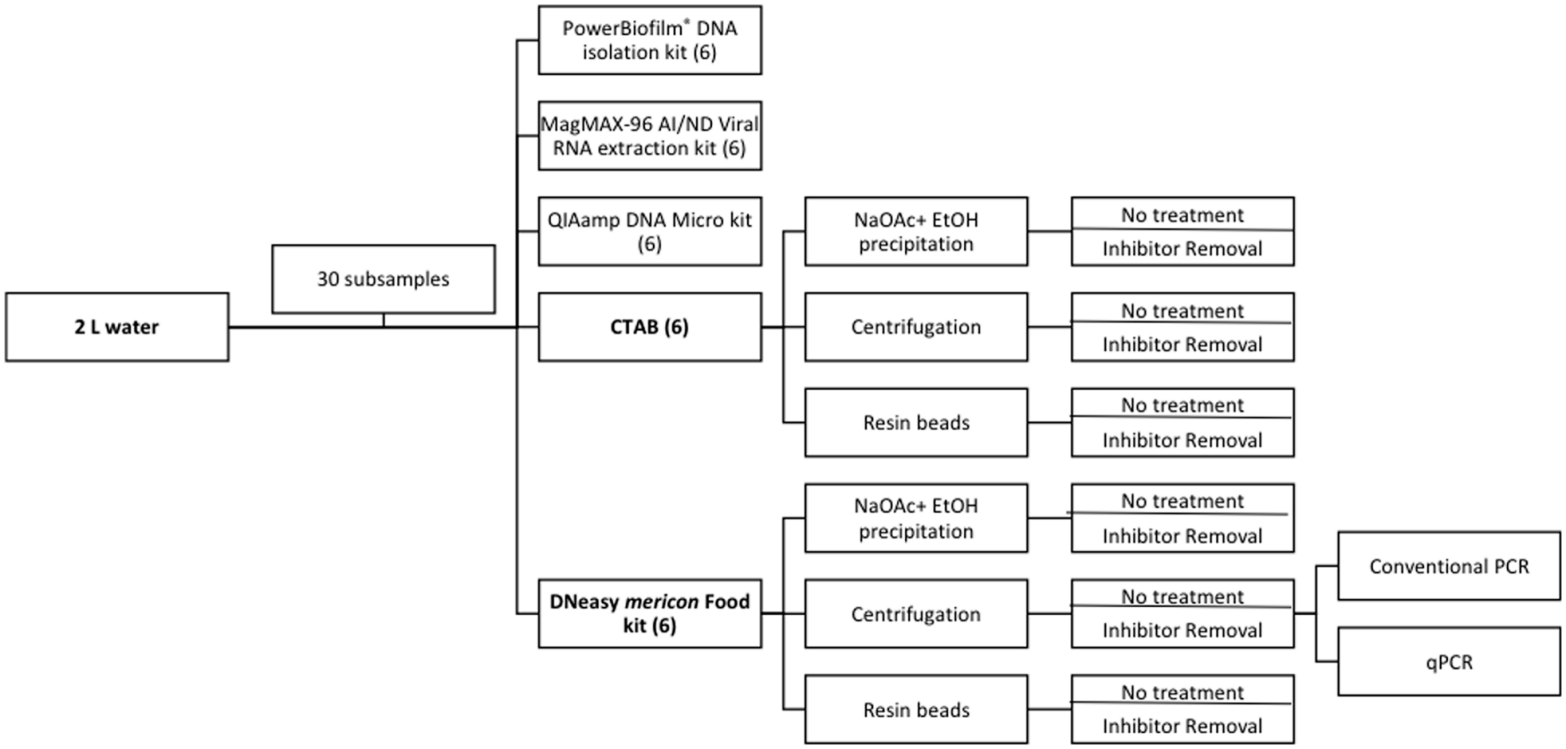
Schematic for the study design of our eDNA capture optimization experiment.

One of our goals was to test various purification techniques in combination with three different capture methods. To reduce the total number of combinations to be tested, we first used a concentration technique (sodium acetate and ethanol precipitation) that was effective in other studies [2, 18, 33] along with 5 extraction methods (detailed below, Fig 2); we selected the top two best-performing techniques. We evaluated these two techniques (detailed below) in combination with two additional capture methods (three total) to identify the overall best combination for the capture and extraction.

For each of two collection dates, 2L of water were collected from a 95L tub serving as a waterer for a single pig. The 2L of water was subsampled and randomly assigned to an extraction kit. Each subsample was aliquoted into three replicates of 15 mL sample water, extracted, and run in triplicate on conventional PCR. Next, the DNeasy *mericon* Food kit and CTAB protocol were compared with various concentration techniques. A second sample collection of 1L of water was collected from the same pig waterer. The 1L sample was subsampled and randomly assigned to a concentration and extraction method combination. After analyzing concentration and extraction success with conventional PCR for each sample, the samples were cleaned with a Zymo IRT column and reanalyzed. The samples that were passed through the IRT columns from the centrifugation and DNeasy *mericon* Food kit extraction combination were used to determine sensitivity of each PCR instrument (conventional PCR and quantitative PCR).

#### Sampling

Water was collected from a 95 L tub that served as a water source for a single female feral pig in captivity at the USDA-APHIS/Colorado State University Wildlife Research Facility. Water was collected on March 3, 2015 and again on March 25, 2015 by submerging a single sterilized 2L Nalgene^®^ bottle, approximately 10 cm below the surface of the water, and filling it. Water was murky and turbid.

The sample was mixed using a magnetic stir bar and plate and then subsampled into thirty 50 mL falcon tubes. Subsamples were numbered in order of collection and then randomly assigned to each of the five extraction techniques using a random number generator. These subsamples were stored for two nights at -80°C. To monitor for contamination, we included one negative control, containing only extraction reagents, with each set of extractions for all methods.

#### Extraction techniques

All eDNA capture and extraction steps were performed within a 12-hour period two days after the initial collection event. Each 50 mL subsample was aliquoted into three replicates of 15 mL each. Aliquots were concentrated by sodium acetate/ethanol precipitation (addition of 1.5 mL of sodium acetate and 33 mL of absolute ethanol to 15 mL sample) [33] with a centrifugation modification (3220 g for 45 minutes) [18]. Sodium acetate and ethanol were added to each of the aliquots and stored overnight at -20°C and then centrifuged [39]. The solution was decanted and the resulting pellet was dried for 5 minutes by inverting the 50 mL tubes [18]. We did not do the additional ethanol wash of the pellet.

Extraction methods that were compared included the MagMAX-96 AI/ND Viral RNA extraction kit (Applied Biosystems, Foster City, California), the QIAamp DNA Micro kit (Qiagen, Hilden, Germany), the CTAB (cetyltrimethyl ammonium bromide) protocol [39, 50], the DNeasy *mericon* Food kit (Qiagen), and the PowerBiofilm^®^ DNA isolation kit (MoBio, Carlsbad, California). The MagMAX-96 AI/ND Viral RNA extraction kit is a magnetic bead/total nucleic acid extraction method. For this kit, the pellet was resuspended in 200 μL of distilled water and extraction was performed via an automated robot (King Fisher 96 Extraction Robot). For the QIAamp DNA Micro kit, the pellet was resuspended in 300 μL of Qiagen buffer ATL and extracted according to protocol on a QIAcube robot (Qiagen) programmed to follow the Forensic Case Work Samples protocol [18]. The CTAB protocol for extraction of aqueous eDNA precipitated/pelleted from water was followed with some modifications [39]. Recipes for the CTAB extraction buffer, Sevag, and LoTE buffer were used from Turner et al. 2014. The pellet/CTAB/Sevag mixture was centrifuged at 3250g instead of 3220g due to centrifuge speed limitations. The DNA pellet was air dried in tissue covered tubes overnight (Kimtech, Pleasant Prairie, Wisconsin) to ensure that all ethanol from the previous step evaporated. The pellet was resuspended in Lote buffer according to Turner’s protocol [39]. The standard protocol for 200 mg of sample was used for the DNeasy *mericon* Food kit. For the PowerBiofilm^®^ DNA isolation kit the pellet was resuspended after concentration in 350 μL of BF 1 solution and moved into extraction; we used a vortex adapter for the bead beating step.

All extracted DNA from each of the five techniques were stored in a -80°C freezer until PCR analysis. For this portion of the study, we used conventional PCR (cPCR) and fragment analysis on an ABI 3130 (Life Technologies) to test for successful feral swine DNA capture and purification. Conventional PCR (cPCR) was chosen as the method of confirmation of successful amplification and comparison of DNA capture and purification because previous eDNA studies show this is a viable method [2, 18] and prior to this study this was the standard method in our lab for confirming target eDNA amplification [18].

Primers were designed using AlleleID (ver. 7.0; Premier Biosoft) for a target fragment of the mitochondrial D-loop region of *Sus scrofa* (NC00845, BLAST) for 101 basepairs (bp) in length (*SusScrofa*DF 5’ CAAGCATTCCATTCGTATG; *SusScrofa*DR 5’ CGCATATTTGTATGTTTGTG). After the primers were designed, we performed a BLAST search to assess specificity using the National Center for Biotechnology Information website [51]. Primers were also tested in the lab for cross-reactivity with DNA extracted from sheep, cow, deer, and dog tissue using the DNeasy Blood & Tissue kit (Qiagen). The optimized assay was used to determine if DNA from these tissues were amplified with the primers, *SusScrofa*DF was labeled with fluorescent dye 6-FAM for visualization of fragments on an ABI3130xl genetic analyzer (Life Technologies, Carlsbad, California).

Each cPCR was a 25 μL reaction containing 2.5 μL 10x Amplitaq buffer (Life Technologies), 0.5 μL MgCl_2_, 1 μL dNTP, 5% DMSO, 0.25 μL of each primer (10μM), 0.25 μL Amplitaq (Life Technologies), 18 μL distilled water, and 1 μL of DNA extract. The thermocycling program optimized for amplification involved 15 min at 95°C; 60 cycles of 30s at 94°C, 45s at 54°C, 1 min at 72°C; and the final extension time was 7 min at 72°C. Each PCR run included a negative control and extraction negative control to monitor for contamination and ensure false positives were not occurring with 60 cycles of amplification. For amplification, each extracted water sample was replicated three times to account for heterogeneity associated with low quality/low quantity DNA assays [2, 52]. Fragment analysis was accomplished on an ABI3130xl genetic analyzer with a Liz500 size standard. A bin was created in Gene Mapper 4.0 (Life Technologies) to identify the target peak at 96 bp. This shift from the 101 bp target amplicon was likely due to the fluorescent dye label on the forward primer causing a mobility shift of the amplified fragment on the analyzer [53, 54]. A positive detection from a water sample was confirmed if at least one out of the three PCR replicates was positive.

### eDNA capture and inhibitor removal

#### Sampling

Water was collected from the same 95 L tub-pig system as used in our previous step. Water was collected on May 6, 2015 by submerging a single sterilized 2L Nalgene^®^ bottle in the tub and filling it. Water quality was similar to previous sampling with suspended particulates causing turbidity.

The water was homogenized and subsampled for a total of sixty 15 mL subsamples. Subsamples were numbered in order of collection and randomly assigned to 6 different treatments using a random number generator.

#### eDNA capture

To determine the most efficient capture and purification combination, the best two extraction techniques (CTAB and DNeasy *mericon* Food kit; Fig 2) were paired with three different concentration techniques. The 6 concentration-purification combinations were as follows: 1) centrifugation-CTAB, 2) sodium acetate/ethanol-CTAB, 3) resin bead- CTAB, 4) centrifugation-DNeasy *mericon* Food kit, 5) sodium acetate/ethanol -DNeasy *mericon* Food kit, and 6) resin bead-DNeasy *mericon* Food kit (Fig 2). We included a negative control, with extraction reagents only, with each set of extractions for all treatments to monitor for contamination.

For concentration by centrifugation, Caldwell et al.’s protocol (2007) of spinning 15 ml samples at 9000g for 15 minutes at room temperature was followed with a modification of resuspending the pellet in the appropriate lysis buffer after decanting the supernatant.

Concentration with resin beads involved adding 0.25 g of Amberlite IRA-900 anion exchange resin (Sigma-Aldrich, St. Louis, Missouri) to each of the 15 mL subsamples and shaking for 2 hours at room temperature (23°C) [36] on a C24KC Refrigerated Benchtop Incubator Shaker (Eppendorf/New Brunswick Scientific, Hamburg, Germany) at 225 RPM, rapid enough to allow the beads to gently swirl in the water. The water was then decanted and the resin beads were resuspended in the lysis buffer of the appropriate protocol for each treatment. For the CTAB extraction, the resin beads were treated as if they were a pellet and the beads in lysis buffer were incubated according to protocol [36]. For the DNeasy *mericon* Food kit, the resin beads were treated as if they were the starting food material. After resuspending the resin beads in the food lysis buffer, everything was transferred to a 2 mL tube and the rest of the manufacturer’s protocol was followed (Qiagen).

The sodium acetate and ethanol precipitation followed the protocol [33] previously described with a modification of eliminating overnight incubation of the mixture. The pellets were dried for 5 minutes after decanting the supernatant [18].

Any additional modifications used for the CTAB protocol and the DNeasy *mericon* Food kit are included in the previous section describing extraction methods. All DNA extractions for each treatment were carried out on the same day of sample collection. Purified DNA from each sample was analyzed using the cPCR amplification and fragment analysis as above. These 60 purified DNA samples were also used to assess whether inhibitor removal would increase sensitivity.

#### Inhibitor removal

Samples from the 6 capture/purification treatments were also run through an inhibitor removal treatment (IRT) column (Zymo Technology) to determine if inhibitors were affecting PCR performance. Conventional PCR and fragment analysis as described previously were used for amplification and visualization. Findings were compared to the results generated without the IRT column treatment.

### Conventional or quantitative PCR?

To compare sensitivity of conventional PCR (cPCR) to real-time quantitative PCR (qPCR), serial dilutions were made from 80% of the optimized protocol extractions (Centrifugation, DNeasy mericon Food kit, Zymo IRT), and then amplified using both cPCR and qPCR protocols. The cPCR program and recipe described above were used with the modification of adding 5 μL of DNA as PCR template to keep the amount of DNA consistent for both PCR assay types (water volume adjusted to 14 μL). The primers described earlier were imported into AlleleID (ver. 7.0; Premier Biosoft) to create a compatible Taqman probe (5’-/56-FAM/AAACCAAAACGCCAAGTACTTAATTAC/3BHQ_1/-3’) with a FAM label and Black Hole Quencher Dye (IDT) for qPCR. Each qPCR reaction was a 30 μL reaction containing 15 μL Taqman environmental mastermix (Life Technology), 1 μL of each primer (10 μM), 1 μL of the probe (2.5 μM), 1 μL BSA, 6 μL distilled water, and 5 μL of DNA extract run on a Biorad real time PCR thermocycler (Biorad, Hercules, CA). The optimized real time thermocycling program involved 10 min at 95°C, 50 cycles of 95°C for 15 sec, and 1 min at 52°C. For our standard curve, we used a synthetic sequence of our target amplicon with additional bases from the pig D-loop sequence to reach the minimum amplicon length (125 bases) for double stranded fragment production (gBlocks Gene Fragments, IDT). We developed a standard curve from 1:10 serial dilutions of this synthetic fragment (1x10^5^ copies/μL to 10 copies/ μL) to evaluate our qPCR assay. We determined our LOQ to be the lowest dilution in which 8 qPCR replicates amplified and our LOD as the standard dilution that was 10-fold below the LOQ (E = 95.1%, R^2^ = 0.992, Slope = -3.446) [55]. Our limit of detection (LOD) was 1 DNA copy/ μL.

Each cPCR and qPCR set included a “no template” negative control with just PCR reagents and the extraction negative controls to monitor for contamination. Each extracted water sample was replicated in qPCR and cPCR three times. The PCR approach that successfully amplified DNA to the lowest dilution was determined to be the most sensitive.

### Statistical analysis

Detection of species using eDNA is likely imperfect [56]; occupancy modeling approaches account for imperfect detection in estimating whether or not sites are “occupied” or used by a species [57]. We used an occupancy approach to estimate the probability of detection (p) for each method in each optimization step: extraction, concentration, and inhibitor removal. Encounter histories were constructed from the set of three PCR replicates where cPCR results were coded as ‘1’ for a positive detection if a peak was observed on the genetic analyzer at the appropriate size (accounting for dye shift) and ‘0’ otherwise. Similarly, qPCR reactions were considered positive if the sample was measured as having a DNA concentration above our LOD, >1 copy/ μL, (coded as ‘1’) and negative (‘0’) otherwise. In all analyses, occupancy was set as a fixed parameter with a value of 1.0 because all water samples were exposed to pigs and “occupied” by pig eDNA. Data was analyzed using the occupancy approach with detection <1 as implemented in Program MARK [58]. Candidate models in each model set were ranked by their AICc values [57] and estimates of the probability of detection reported here come from the top-ranked model from each set.

We developed an *a priori* candidate set of occupancy models for the extraction method analysis; this set included models incorporating the effect of extraction method, sampling date, and the additive combination of the two. Sampling date was included because differences in water quality or amount of eDNA shed into the water on each date could affect the probability of detection. The *a priori* set of candidate models for optimal concentration and inhibitor removal method included models with the method of extraction, concentration method, inhibitor removal, and the additive combination of all three factors on the probability of detection of eDNA.

For all optimization steps, the single method with the highest probability of detection (p) was carried on to the next step of optimization. However, we carried over the top two purification (extraction) techniques (DNeasy *mericon* Food kit and CTAB) into the concentration/inhibitor removal optimization step (Fig 2). For the analysis comparing the sensitivity of cPCR with qPCR, the *a priori* set of candidate models included models with an effect of the dilution of the eDNA extraction, the amplification method, and the additive combination of the two. Finally, the results of the resin bead pilot study are portrayed as the proportion of qPCR positive detections for each of the three 10 L water samples.

### Sequence verification

Twenty percent of PCR products from the optimized eDNA capture method that produced a positive detection were sequenced for verification that the target sequence was amplified. Sequencing of PCR products was completed using ABI BigDye chemistry (Life Technologies). Cycle sequencing purification was accomplished through the PrepEase (USB) protocol. Sequences were imported into Sequencher for raw sequence clean up (ver.5.1; Gene Codes Corp.) and a BLAST search was performed using the National Center for Biotechnology Information database to ensure the amplicon was in fact pig [51].

## Results

### eDNA purification (extraction)

Extraction performance varied between the two collection dates (March 3, 2015 and March 25, 2015). For the first collection date, all methods except the MagMAX-96 AI/ND Viral RNA extraction kit produced at least one positive detection. For the second collection, only two methods (CTAB and DNeasy *mericon* Food kit) produced any positive detections.

The best supported model for eDNA purification method included the effects of collection date and extraction method, carrying almost all of the Akaike weight (*ω*_i_ = 0.99) (Table 1). The probability of detection (p) of eDNA was highest for the CTAB extraction protocol (Collection 1: 0.222, Collection 2: 0.074) and DNeasy *mericon* Food kit (Collection 1: 0.222, Collection 2: 0.019) so these methods were carried into the next step of optimization (Table 2). The probabilities of detection for both the CTAB extraction method and DNeasy *mericon* Food kit were lower from the second collection date than the first (Table 2).

**Table 1:** Model ranking of occupancy models evaluating the probability of detecting eDNA from turbid water using various DNA extraction techniques (“extraction”) and the effect of water collection date.

Model	K	AICc	Delta AICc	Akaike weight	Deviance
p(extraction+date)	10	254.576	0.000	0.99998	48.967
p(date)	2	276.858	22.282	0.00001	88.483
p(extraction)	5	283.133	28.557	0.00000	88.481

The number of parameters (K) in each model, the small sample sized-corrected AIC values (AICc), the AICc differences (delta AICc), the Akaike weight for each model, and deviance are reported for each candidate model.

**Table 2:** The probability of detection of eDNA estimated based on the best model (Table 1) of collection date and extraction method.

Collection Period and Extraction Method	Probability of Detection (p)
Period 1 CTAB	0.222
Period 1 QIAamp DNA Micro kit	0.222
Period 1 DNeasy *mericon* Food kit	0.222
Period 1MagMAX-96 AI/ND Viral RNA	0.000
Period 1 PowerBiofilm^®^	0.056
Period 2 CTAB	0.074
Period 2 QIAamp DNA Micro kit	0.000
Period 2 DNeasy *mericon* Food kit	0.019
Period 2 MagMAX-96 AI/ND Viral RNA	0.000
Period 2 PowerBiofilm^®^	0.000

The CTAB protocol and DNeasy mericon Food kit were the only extraction methods that produced positive detections in both water collection events.

### eDNA capture and inhibitor removal

The best supported model, with an Akaike weight of 1.0, for concentration technique and inhibitor removal was the model with the additive combination of concentration, extraction, and inhibitor removal (Table 3). Detection varied across concentration and extraction methods and was overall higher when inhibitor removal techniques were used (Table 4). The highest probability of detection was 0.70; the combination of techniques leading to this level of detection was centrifugation, DNeasy *mericon* Food kit, and Zymo IRT column inhibitor removal (Table 4).

**Table 3:** Model ranking of occupancy models evaluating the probability of detecting eDNA from turbid water using DNA concentration, extraction technique, and inhibitor removal (“IRT”).

Model	K	AICc	Delta AIC	Akaike weight	Deviance
p(concentration+extraction+IRT)	12	215.770	0.000	1.000	58.485
p(concentration)	3	310.603	94.834	0.000	174.028
p(extraction)	2	320.664	104.894	0.000	186.192
p(IRT)	2	327.275	111.505	0.000	192.803

**Table 4:** The probability of detection estimated based on the best model: P(concentration+extraction+IRT).

Concentration + Extraction + Inhibitor Removal Models	Probability of Detection (p)
Cent + CTAB + NT	0.000
Cent + Food + NT	0.100
Resin + CTAB + NT	0.000
Resin + Food + NT	0.000
NaOAc/EtOH+CTAB + NT	0.000
NaOAc/EtOH + Food + NT	0.400
Cent + CTAB + IRT	0.367
**Cent + Food + IRT**	**0.700**
Resin + CTAB + IRT	0.067
Resin + Food + IRT	0.000
NaOAc/EtOH+CTAB + IRT	0.000
NaOAc/EtOH + Food + IRT	0.667

Bolded is the highest probability of detection through concentration by centrifugation, extraction by DNeasy mericon Food kit, and IRT treatment. (NT: No treatment; IRT: Inhibitor Removal Technology)

### Conventional or quantitative PCR?

**Fig 3:**
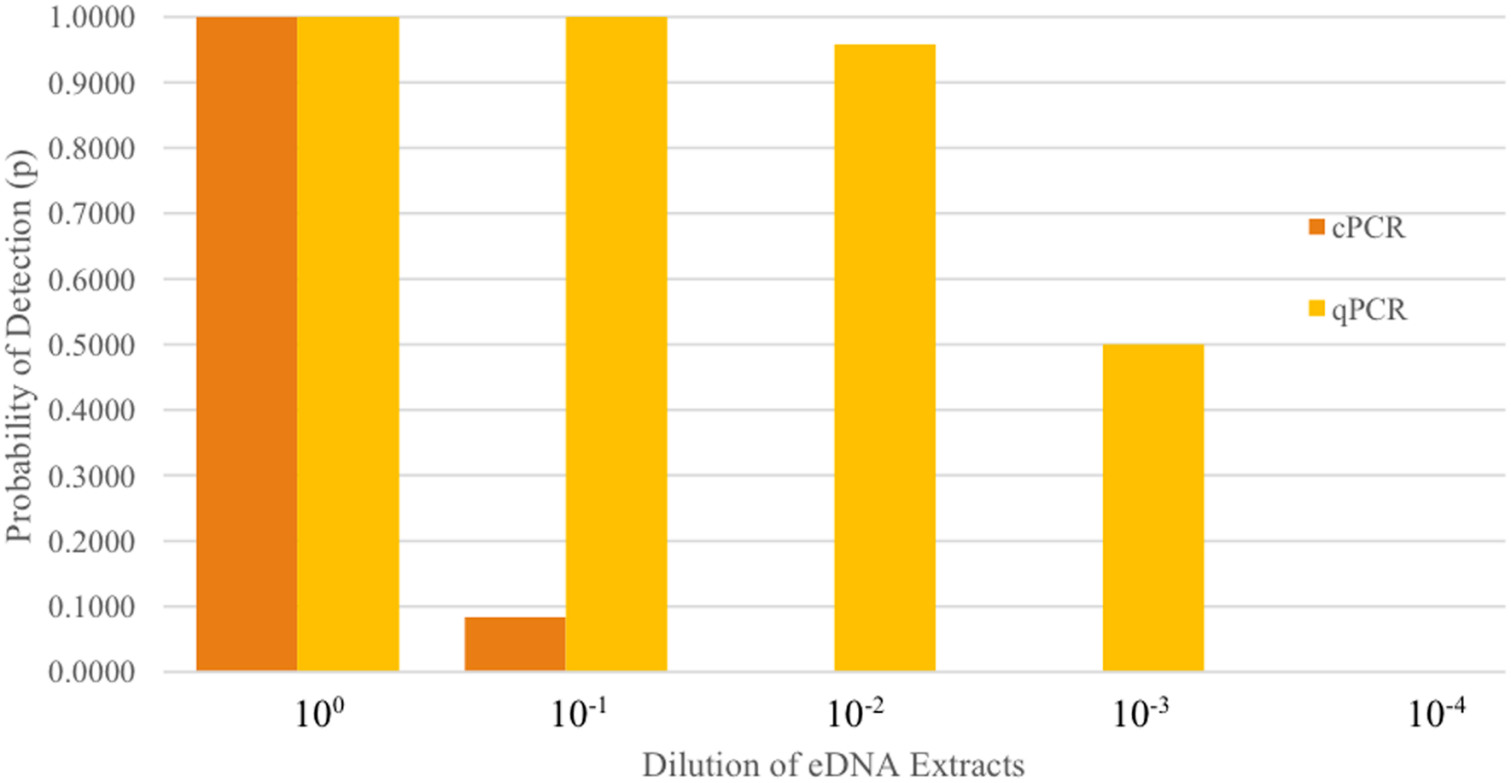
Sensitivity, or the ability to detect low levels of eDNA, with cPCR and qPCR amplification methods across dilutions of eDNA samples.

No cross reactivity occurred when testing our assay on tissue samples from non-target species. Detection probabilities, as a measure of assay “efficiency”, were compared between cPCR with fragment analysis and qPCR to assess the sensitivity of each to detect low concentrations of eDNA. The best supported model, with an Akaike weight of 1.0, was the model with the additive combination of the amplification method (cPCR vs. qPCR) and the level of eDNA extraction dilution (Table 5). Our qPCR runs performed within acceptable ranges of efficiency (between 90%-110%), with slope between -3.1 and -3.6, and R^2^ >0.99. Both cPCR and qPCR performed equally well on extractions when the template DNA was 5 μL. However, qPCR was more sensitive than cPCR when the DNA extraction was diluted. Conventional PCR lost sensitivity (i.e., fewer positive detections) as early as the 10^−1^ dilution. Quantitative PCR retained perfect detection until the 10^−2^ dilution (Fig 3).

**Table 5:** Model ranking of occupancy models evaluating the probability of detecting eDNA from turbid water with two amplification methods (cPCR and qPCR).

Model	K	AICc	Delta AIC	Akaike weight	Deviance
p(PCR method+dilution)	10	78.541	0.000	1.000	25.981
P(dilution)	5	197.462	118.921	0.000	157.280
P(PCR method)	2	277.856	199.315	0.000	244.328

The additive model of both the PCR method and dilution was used to estimate the probability of detection for each eDNA dilution.

Fragment analysis on cPCR lost sensitivity at the first dilution. Real time qPCR provided positive detection consistently until the 10^−2^ dilution where it then gradually lost sensitivity.

### Sequencing verification

The randomly selected PCR products were all confirmed as a portion of the D-loop region of *Sus scrofa*.

## Discussion

Our data clearly show that techniques in concentration, extraction, and amplification of eDNA have varying efficiencies in detecting terrestrial vertebrate eDNA from turbid water samples. Our optimal protocol for eDNA detection from turbid water samples is to concentrate a 15 mL water sample by centrifugation, extract the eDNA using the DNeasy *mericon* Food kit, follow up with Zymo IRT columns to remove any inhibitors, and amplify the DNA with our qPCR assay.

In the first step of optimization, the two sample collection dates varied in detection probability; this is likely due to differences in water quality at the two times of collection. The water in the first collection may have had fewer inhibitors, perhaps more DNA was shed by the pig, or environmental conditions varied enough between the two collection dates to cause a difference in eDNA detectability.

Both the CTAB and DNeasy *mericon* Food kit are chloroform-based extraction methods and these showed the most promise purifying eDNA samples, in keeping with several other studies [37–39, 59, 60]. Though the DNeasy *mericon* Food kit has not yet been tested on eDNA to our knowledge, this kit performed well with fecal samples that likely also contained inhibitors prior to extraction [61]. Both protocols use CTAB which can either form complexes with nucleic acids in low salt conditions or with cellular inhibitors in high salt conditions. We carried both of these methods forward given their consistent performance across both water collection periods.

Prior to inhibitor removal with Zymo IRT columns, it appeared that the addition of sodium acetate with ethanol precipitation paired with the DNeasy *mericon* Food kit extraction provided the optimal method for eDNA capture. However, after inhibitor removal, the combination of centrifugation concentration and DNeasy *mericon* Food kit extraction had a slightly higher probability of detection (Table 4). One drawback to centrifugation using 50 mL falcon tubes is the need for a centrifuge that can accommodate them. We selected a fixed angle rotor that could hold 12 falcon tubes (50 mL) and spin at the required 9000 x g (Beckman); most large rotors are swing style and cannot spin above 3220 x g. Because the sodium acetate/ethanol precipitation concentration technique performed nearly as well as centrifugation, this method may be an acceptable alternative when an appropriate centrifuge is not available.

We recommend using a post-extraction inhibitor removal step. Our results show a strong effect of the Zymo IRT columns in cleaning up elutions as shown by a higher probability of eDNA detection when this step was included (Table 4). This is most obvious for the centrifugation concentration and DNeasy *mericon* Food kit combination: the probability of detection increased from 0.40 without inhibitor removal to 0.70 with inhibitor removal (Table 4) when the cPCR assay involved 1 μL DNA template. In some cases, eDNA was only detected after inhibitor removal (e.g., centrifugation concentration and CTAB extraction); this is most likely due to the inherent load of inhibitors in muddy, turbid water. Though we did not test qPCR performance on untreated inhibited samples, an ongoing study involving environmental samples collected from water bodies in Texas shows that Zymo Inhibitor Removal Treatment does affect detectability of eDNA (unpublished data). In some samples DNA was not detected until after Zymo treatment while in others, DNA was lost in the treatment and not detected post Zymo treatment. Therefore, each study should assess the utility of the Zymo treatment for their samples.

We also recommend using qPCR as the amplification method for eDNA detection unless a lab only has cPCR. While both techniques we examined produced a probability of detection of 1.0 when we used 5 μL of template eDNA, there is a clear difference in sensitivity when DNA is diluted. The difference in sensitivity between cPCR and qPCR could be due to the instrument performance or the reagents used as different reagents are used for each amplification method (i.e. PCR mastermix differences and inclusion of a probe with qPCR).

Samples collected in the waterers may have a wide range of eDNA availability (i.e., concentration) due to stochasticity or eDNA clumping [62] in our environmental samples, despite the effort to homogenize. This finding suggests that further optimization in sampling scheme should occur before applying this test in the field for management questions.

We did not successfully capture eDNA from 15 mL water samples using the resin bead concentration method. Nevertheless, once we increased the volume of sample water to 10L, resin beads showed promise in capturing eDNA (S1 File, S1 Table). A suggestion to improve this concentration technique is to use a filter or screen to separate the resin beads from debris at the transfer step to reduce potential inhibitor material. Resin beads may also get oversaturated with non-target DNA before binding to target eDNA.

Factors that could influence the ability to detect pig eDNA using our assay may include the level of turbidity (inhibitors), amount of eDNA shed into the system, or environmental factors. In order for an assay to be useful, its limits of detection should be explored, as we have done here. By comparing the probability of detection for each step in optimization, a robust method of eDNA capture in our turbid water system was established. The sample collection, concentration, purification, inhibitor removal, and amplification steps can be completed all within a single day, which allows for a fast turn-around time when presented with a pressing wildlife conservation or management question.

While comparing detection probabilities was the primary goal of this study, we will also compare cost and feasibility. With the optimized method, a single water sample extracted in triplicate, cleaned with the Zymo IRT columns, and run in triplicate with qPCR costs $34.98. One disadvantage of the optimized protocol is that the centrifugation speed and water volume required the use of an ultra centrifuge (Beckman) that could spin 50 ml tubes. This limits the number of samples that can be processed. The sodium acetate and ethanol concentration method performed nearly as well as centrifugation and does not require an ultra centrifuge or a fume hood. If a laboratory is not equipped with a centrifuge and fixed angle rotor capable of spinning 50 ml tubes at to 9000g, sodium acetate and ethanol precipitation could be an alternative. While the CTAB method was much cheaper, the probability of detection was lower and it was more difficult to carry out in the lab compared to the DNeasy mericon Food kit (i.e. Sevag use with CTAB); though chloroform is still required for the DNeasy mericon Food kit and thus involves a fume hood for safety. The costs of each extraction method are listed in S2 Table.

Here, we developed a complete, optimized method for detection of eDNA from samples collected from a turbid water system. We show dramatic differences in the probability of detection with each option for each step of eDNA capture and amplification. Comparing efficiencies of concentration, extraction, inhibitor removal post-extraction, and amplification techniques can reduce biases in capturing and detecting eDNA [4, 41]. Understanding the biology of the system from which eDNA is collected is critical as it should influence the choices made for each step of the process.

## Supporting information

S1 File Resin bead pilot study. Summary of a small experiment to test if resin beads could be an effective method of eDNA capture from larger volumes of turbid water.
(DOCX)

S1 Table Detection probabilities for eDNA samples concentrated using resin beads in three 10L samples of turbid water. Samples were extracted using CTAB method followed by cleaning the elutions with inhibitor removal technology and amplification using qPCR.
(DOCX)

S2 Table Cost per sample for extraction methods compared. (DOCX)
