# Supplementary material for: Clearing muddied waters: Capture of environmental DNA from turbid waters
Source: PLoS One. 2017 Jul 7;12(7):e0179282. doi: 10.1371/journal.pone.0179282 (PMC5501390; doi:10.1371/journal.pone.0179282)
Supplement: S1 File — Summary of a small experiment to test if resin beads could be an effective method of eDNA capture from larger volumes of turbid water. (DOCX) [file pone.0179282.s001.docx]

**S1 File:** **Resin bead pilot study**

We performed a small experiment to test if resin beads could be an effective method of eDNA capture from larger volumes of turbid water. We suspected that they may perform better with larger volumes of water than our initial assessment (15 mL) because they have been successfully used in the past to capture virus particles from 50 mL and 1L water samples [[36](#_ENREF_36)]. To test this hypothesis, three 19 L buckets were filled with 10 L of water collected from a 50L tub that served as a waterer for two captive boars. A fourth bucket was filled with 10L of tap water to serve as a negative control. We added 0.5 g of Amberlite resin beads and a magnetic stir bar to each bucket and mixed the water on a stir plate for 2 hours at a rate quick enough to keep the beads afloat and dispersed through the water sample. At the end of two hours, the beads were allowed to settle and the majority of the water was decanted. A small volume of water was left in with the beads and a pipette with a 25 mL pipette tip was used to transfer the beads into a 50 mL falcon tube. The remaining water collected in the transfer of the beads was removed and the DNA on the beads was immediately extracted using the CTAB extraction protocol for extraction of aqueous eDNA precipitated/pelleted from water [[39](#_ENREF_39)] with the modifications described above. The CTAB extraction protocol was chosen because it was the only extraction method that yielded any positive detections with resin bead concentration with the 15 mL sample volume. Each sample was amplified in triplicate with qPCR after inhibitor removal.

We successfully detected eDNA from resin beads when we collected larger amounts of water (10L). The proportion of qPCR positives varied across the three 10L water samples ranging from a probability of detection of 0.00 to 0.67 (S1 Table).
