# Supplementary material for: Clearing muddied waters: Capture of environmental DNA from turbid waters
Source: PLoS One. 2017 Jul 7;12(7):e0179282. doi: 10.1371/journal.pone.0179282 (PMC5501390; doi:10.1371/journal.pone.0179282)
Supplement: S1 Table — Samples were extracted using CTAB method followed by cleaning the elutions with inhibitor removal technology and amplification using qPCR. (DOCX) [file pone.0179282.s002.docx]

**S1 Table: Detection probabilities for eDNA samples concentrated using resin beads in three 10L samples of turbid water.**

| 10 L Bucket ID | Proportion of qPCR positive detections |
| --- | --- |
| 1 | 0.667 |
| 2 | 0.333 |
| 3 | 0.000 |

Samples were extracted using CTAB method followed by cleaning the elutions with inhibitor removal technology and amplification using qPCR.
