# Supplementary material for: Clearing muddied waters: Capture of environmental DNA from turbid waters
Source: PLoS One. 2017 Jul 7;12(7):e0179282. doi: 10.1371/journal.pone.0179282 (PMC5501390; doi:10.1371/journal.pone.0179282)
Supplement: S2 Table — (DOCX) [file pone.0179282.s003.docx]

**S2 Table: Cost per sample for extraction methods compared.**

| **Extraction Method** | **Cost per Extraction** |
| --- | --- |
| DNeasy Mericon Food Kit | $3.12 |
| Cetyl trimethyl ammonium bromide (CTAB) | $0.20 |
| PowerBiofilm DNA Isolation Kit | $7.80 |
| MagMax 96 AI/ND Viral RNA | $3.55 |
| QIAamp DNA Microkit | $4.54 |
